# Supplementary figures and images for: Receptor Activity-Modifying Protein 2 (RAMP2) alters glucagon receptor trafficking in hepatocytes with functional effects on receptor signalling
Source: Mol Metab. 2021 Jul 13;53:101296. doi: 10.1016/j.molmet.2021.101296 (PMC8363841; doi:10.1016/j.molmet.2021.101296)

Supplementary Figure 1

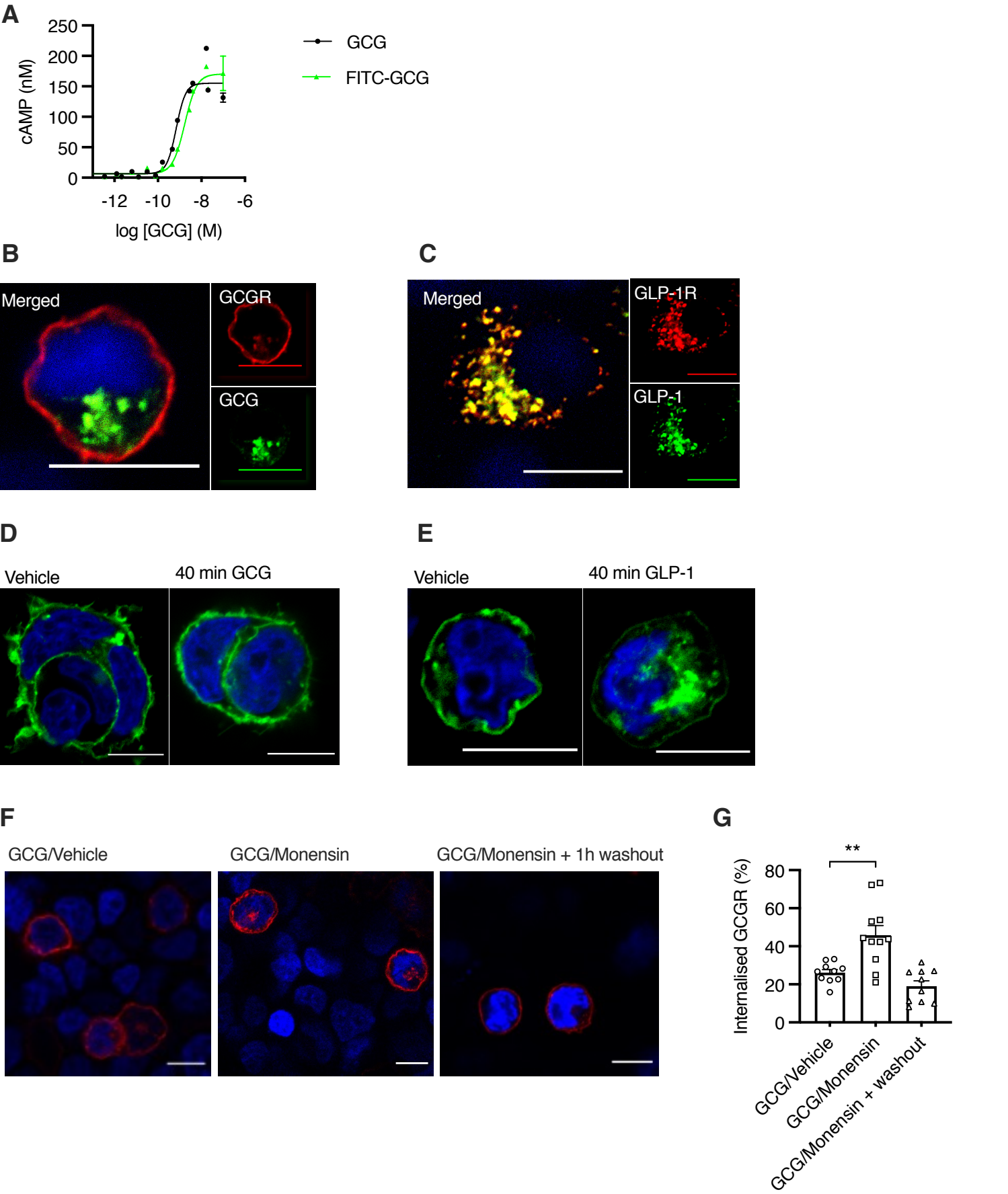

Supplement: Multimedia component 2 — Supplementary Figure 1 – Agonist-stimulated internalisation of GCGR vs. GLP-1R in the rat pancreatic beta cell line INS-1 832/3 and with GFP-tagged receptors in HEK293T cells. A: cAMP dose responses to GCG vs. FITC-GCG in Huh-GCGR cells after 30 minutes stimulation; n=2; 4-parameter fit of pooled data shown. B: INS-1 832/3 cells transfected with SNAP-GCGR (labelled with SNAP-Surface 549 probe, red) and stimulated with FITC-GCG (green). C: INS-1 832/3 cells transfected with SNAP-GLP-1R (labelled with SNAP-Surface 549 probe, red) and stimulated with FITC-GLP-1 (green). D: HEK293T cells transfected with GCGR-GFP (green) with and without GCG stimulation for 40 minutes. E: HEK293T cells transfected with GLP-1R-GFP (green) with and without GLP-1 stimulation for 40 minutes. F: HEK293T cells transfected with SNAP-GCGR (labelled with SNAP-Surface 549 probe, red), stimulated with GCG alone, GCG plus monensin, or GCG plus monensin followed by a 1-hour washout period as indicated. Nuclei stained with DAPI (blue); scale bars = 10 μm. G: Quantification of SNAP-GCGR internalisation from F; internalised GCGR is expressed as % of total; n=10, mean ± SEM; one-way ANOVA with Dunnett’s post-hoc test; ∗∗p<0.01. [file mmc2.pdf]

Supplementary Figure 2

**A**

- RAMP2

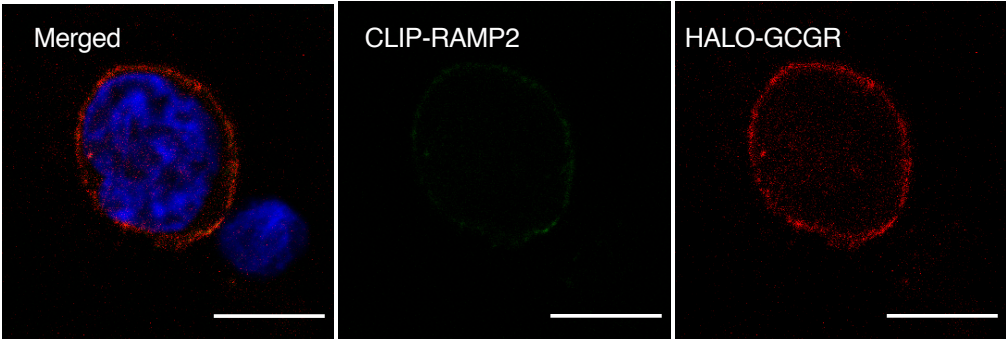

+ RAMP2

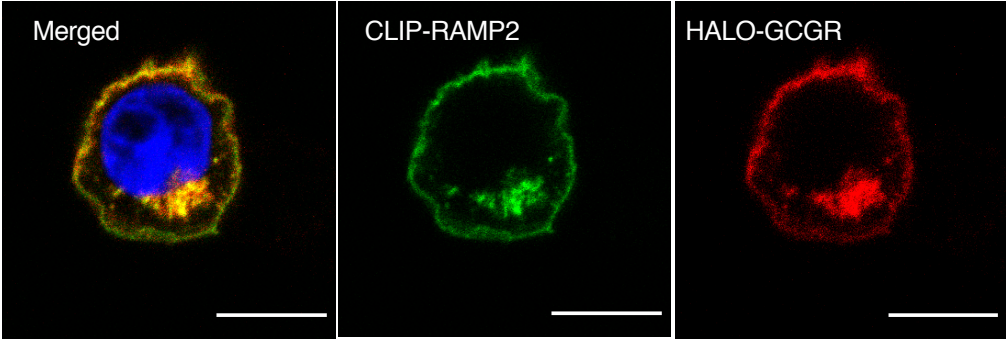

**B**

- RAMP2

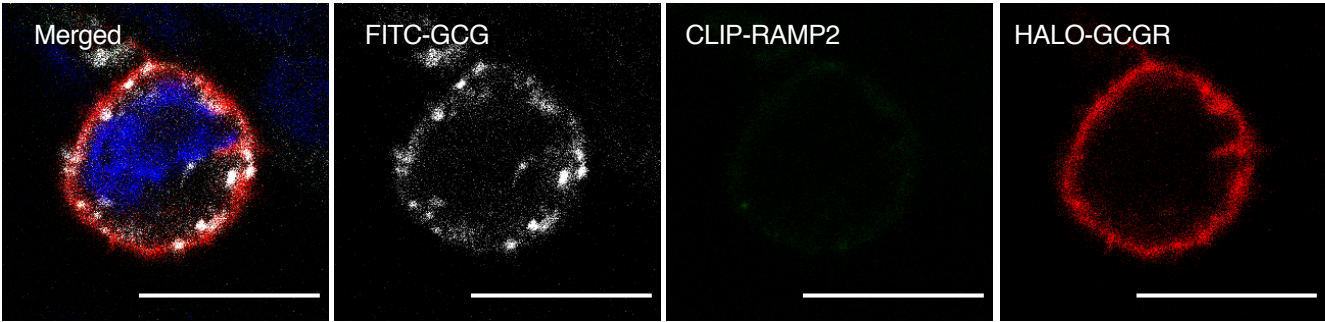

+ RAMP2

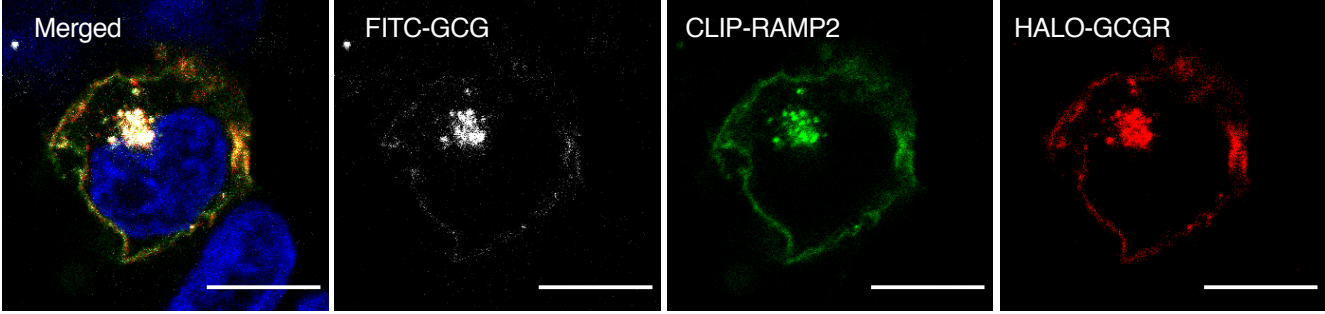

Supplement: Multimedia component 3 — Supplementary Figure 2 – HALO-tagged GCGR is predominantly localised at the cell surface in the absence of RAMP2 but retained intracellularly in the presence of RAMP2. A, B: HEK293T cells transfected with HALO-GCGR (labelled with HALO-AlexaFluor 660 probe, red) with or without co-expressed CLIP-RAMP2 (labelled with CLIP-Surface 547, green) and treated with vehicle (A) or FITC-GCG (grey) (B). Nuclei stained with DAPI (blue); scale bars = 10 μm. [file mmc3.pdf]

Supplementary Figure 4

A

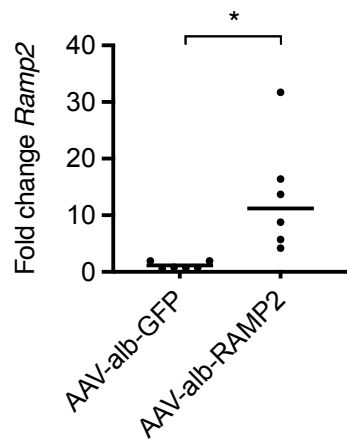

B

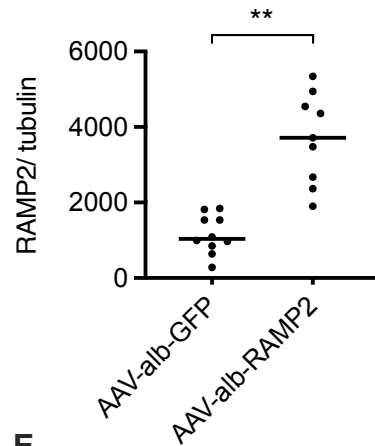

C

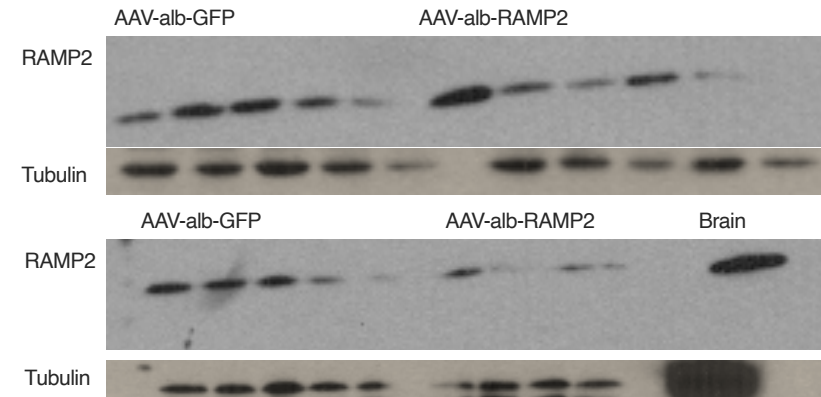

D

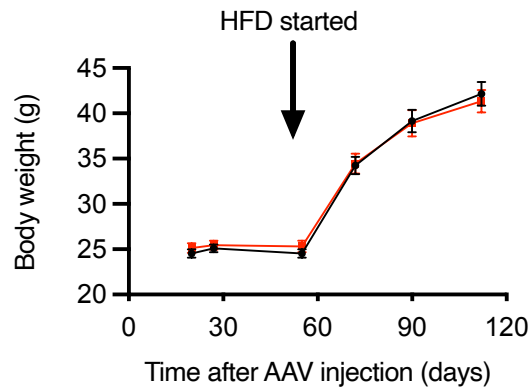

E

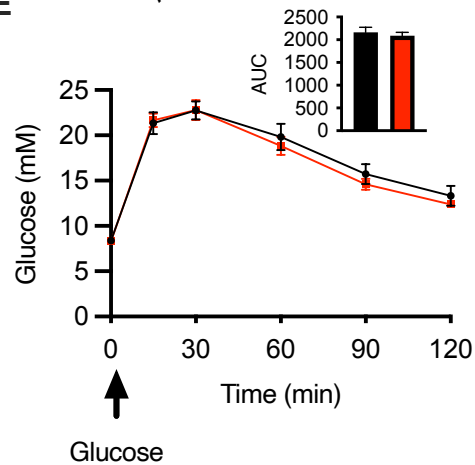

F

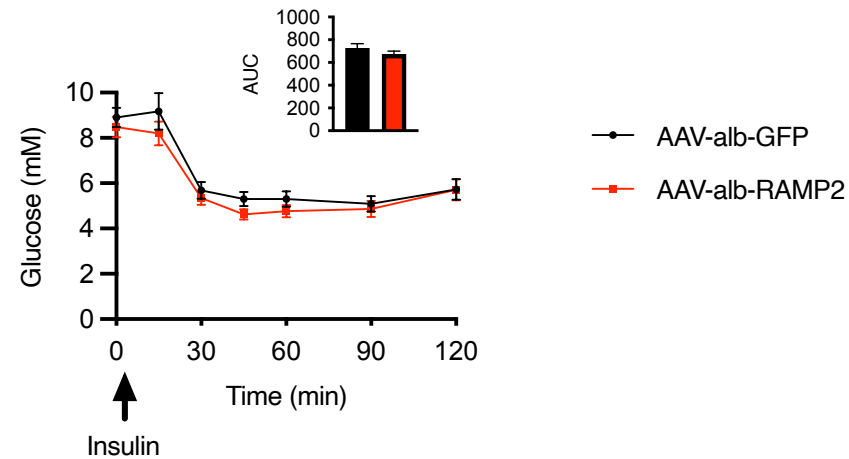

G

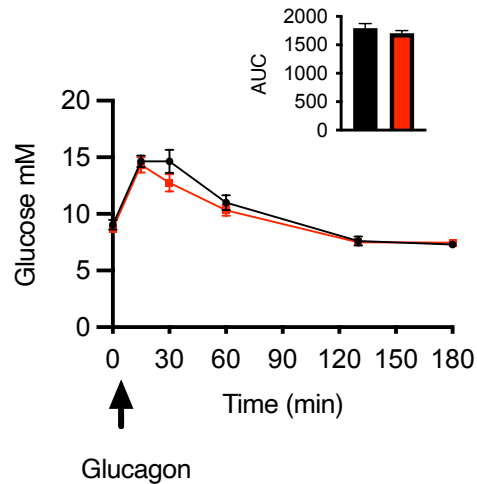

H

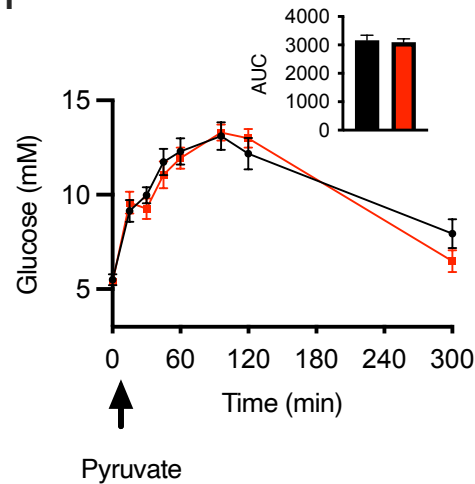

I

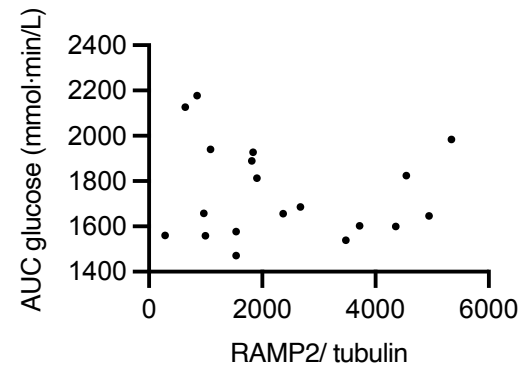

Supplement: Multimedia component 4 — Supplementary Figure 3 – Agonist-stimulation at the GCGR leads to cAMP production and recruitment of β-arrestin-2. A, B: cAMP (A) and β-arrestin-2 (B) dose responses to GCG stimulation in GCGR DiscoverX cells (Eurofins DiscoverX); n=4; data is mean ± SEM; 4-parameter fit of pooled data shown. C, D: Plasma membrane (C) and endosomal (D) baseline Gαs activity measured by NanoBiT complementation assay in the presence of pcDNA3.1 or RAMP2; n=5; paired t-test. E: Representative images from Western blots of SNAP-GCGR and tubulin levels in SNAP-GCGR-expressing HEK293T cells with and without RAMP2 co-expression following stimulation with 100 nM GCG for 4 hours; image of membrane cropped as indicated by black surrounding lines and spliced together (see Figure 3L for quantification). [file mmc4.pdf]

**Supplementary Figure 4**

**A**

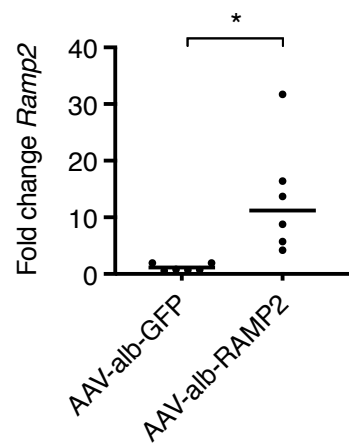

**B**

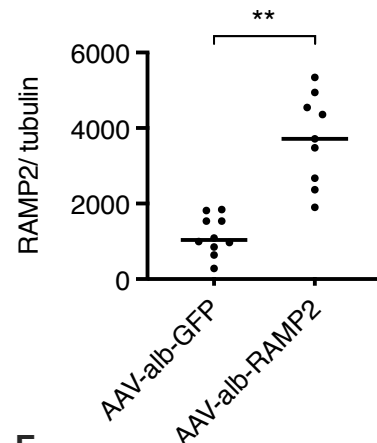

**C**

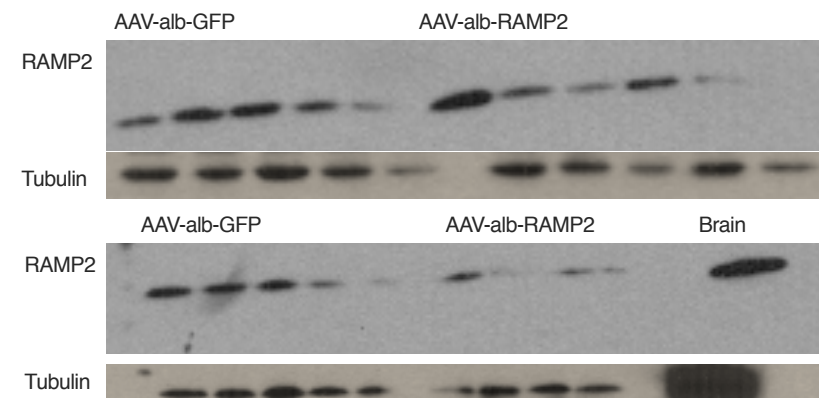

**D**

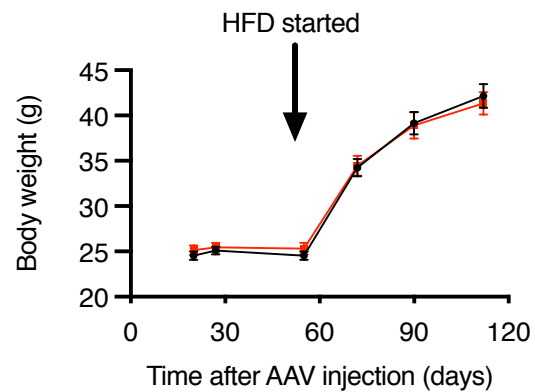

**E**

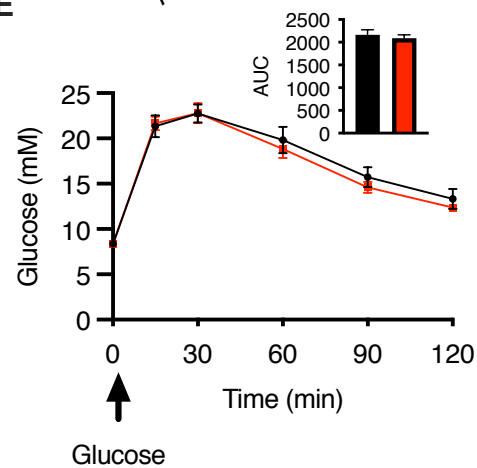

**F**

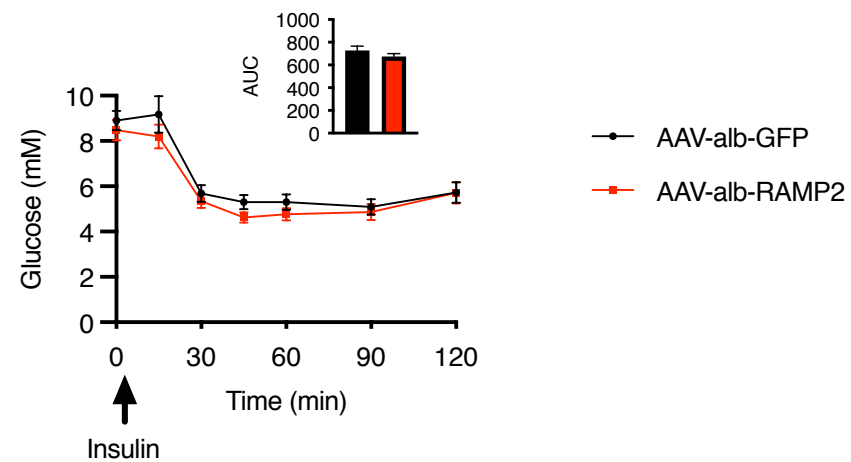

**G**

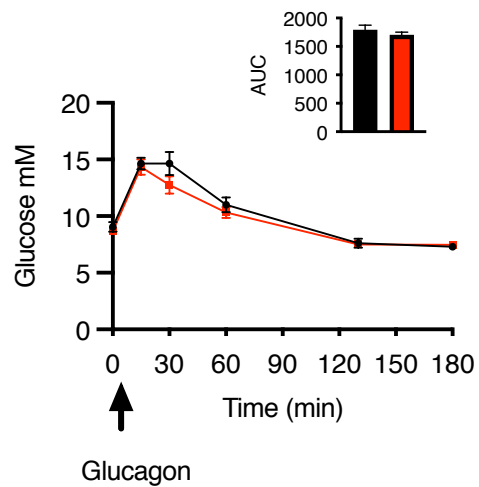

**H**

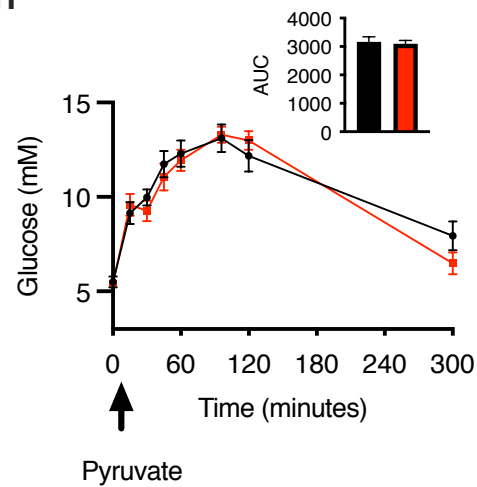

**I**

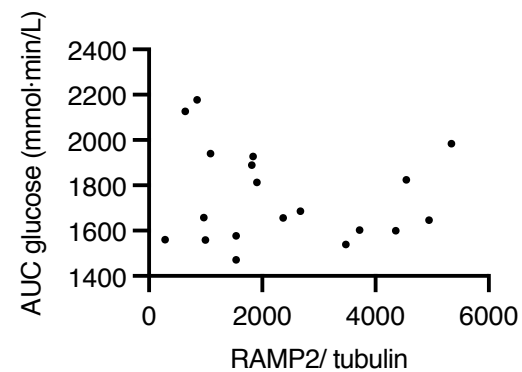

Supplement: Multimedia component 5 — Supplementary Figure 4 – Up-regulation of hepatic RAMP2 in obese adult male mice is not associated with a change in phenotype. A: Ramp2 gene expression normalised to AAV-alb-GFP, data is mean ± SEM; n=4–5 in each group. B: Hepatic RAMP2 protein expression normalised to tubulin; n=9–10 in each group. C: Western blot depicting RAMP2 (upper panel) and tubulin (lower panel) levels from mice 4 months post-AAV injection; AAV-alb-GFP (GFP) or AAV-alb-RAMP2 (R2) as indicated; mouse brain as positive control. Images of membranes have been cropped and spliced together. D: Body weight. E: Glucose tolerance test. F: Insulin tolerance test. G: Glucagon challenge. H: Pyruvate tolerance test. D-H: Experiments performed in mice 3–4 months post-AAV injection, with AUC shown as inset; n=9–10 per group; data is mean ± SEM. I: AUC for GTT vs. protein expression of RAMP2 for each mouse. Statistical significance was analysed using unpaired t-tests for A and B, and AUCs; and two-way ANOVA with Sidak’s multiple comparison test for time-courses; ∗p<0.05 and ∗∗∗∗p<0.0001. [file mmc5.pdf]
